# Lifelong endurance training attenuates age-related genotoxic stress in human skeletal muscle

**DOI:** 10.1186/2046-2395-2-11

**Published:** 2013-07-12

**Authors:** James N Cobley, George K Sakellariou, Scott Murray, Sarah Waldron, Warren Gregson, Jatin G Burniston, James P Morton, Lesley A Iwanejko, Graeme L Close

**Affiliations:** 1 Research Institute for Sport and Exercise Science, Liverpool John Moores University, Liverpool, L3 3AF, UK; 2 Institute of Ageing and Chronic Disease, University of Liverpool, Liverpool, L69 3GA, UK; 3 Cardiology Department, Liverpool Heart and Chest Hospital, Liverpool, L14 3PE, UK; 4 Stepping Hill Hospital, Stockport, SK2 7JE, UK

**Keywords:** PARP-1, Cleaved PARP-1, PARG, Apoptosis, DNA repair, Exercise, Training, Ageing

## Abstract

**Background:**

The aim of the present study was to determine the influence of age and habitual activity level, at rest and following a single bout of high-intensity exercise, on the levels of three proteins poly(ADP-ribose) polymerase-1 (PARP-1), cleaved-PARP-1 and poly(ADP-ribose) glycohydrolase (PARG), involved in the DNA repair and cell death responses to stress and genotoxic insults. Muscle biopsies were obtained from the vastus lateralis of young trained (22 ± 3 years, *n* = 6), young untrained (24 ± 4 years, *n* = 6), old trained (64 ± 3 years, *n* = 6) and old untrained (65 ± 6 years, *n* = 6) healthy males before, immediately after and three days following a high-intensity interval exercise bout.

**Results:**

PARP-1, which catalyzes poly(ADP-ribosyl)ation of proteins and DNA in response to a range of intrinsic and extrinsic stresses, was increased at baseline in old trained and old untrained compared with young trained and young untrained participants (*P* ≤ 0.05). Following exercise, PARP-1 levels remained unchanged in young trained participants, in contrast to old trained and old untrained where levels decreased and young untrained where levels increased (*P* ≤ 0.05). Interestingly, baseline levels of the cleaved PARP-1, a marker of apoptosis, and PARG, responsible for polymer degradation, were both significantly elevated in old untrained compared with old trained, young trained and young untrained (*P* ≤ 0.05). Despite this baseline difference in PARG, there was no change in any group following exercise. There was a non-significant statistical trend (*P* = 0.072) towards increased cleaved-PARP-1 expression post-exercise in younger but not old persons, regardless of training status.

**Conclusions:**

Collectively, these results show that exercise slows the progression towards a chronically stressed state but has no impact on the age-related attenuated response to acute exercise. Our findings provide valuable insight into how habitual exercise training could protect skeletal muscle from chronic damage to macromolecules and may reduce sarcopenia in older people.

## Background

Skeletal muscle undergoes profound age-related deterioration, characterized by striking decreases in muscle mass and function [1,2]. Age-related muscle degeneration can severely affect the health and quality of life of older people, often leading to frailty and leaving them unable to perform everyday tasks and significantly increasing the risk of falls [3]. Although the pathogenesis of age-related muscle deterioration is complex, it is emerging that DNA damage may play an important role, perhaps due to an increase in reactive oxygen (ROS) and reactive nitrogen species (RNS) [4] as a consequence of mitochondrial dysfunction [5].

An imbalance in RNS and ROS leads to both oxidative and nitrative stress, which can result in oxidation or nitration of macromolecules, especially DNA [6], and inflammation [7]. The consequences of DNA damage, that is, genomic rearrangements and mutations, are closely associated with the ageing process [8]. One theory is that mitochondrial dysfunction leads to oxidative stress and can contribute to the ageing process by the accumulation of DNA damage and mutations and via cell depletion, as a consequence of senescence and apoptosis [8]. In support of this, several studies have demonstrated that the oxidative DNA damage adduct 8-oxoguanine (8-oxoGua) is increased in muscle tissue of older people [9,10]. Furthermore, recent evidence connects mitochondrial dysfunction with apoptosis in skeletal muscle suggesting that increased DNA damage with age could promote apoptosis and fiber loss [11,12].

We, and others, have previously shown that many of the adverse outcomes of ageing in skeletal muscle, particularly sedentary ageing, are attenuated and/or reversed by life-long training [13-17]. Although exercise has the capacity to increase acute DNA damage, via elevated ROS production and other homeostatic perturbations [18], the ensuing adaptive response could reduce the accumulation of DNA damage and mutations and thus prevent pro-apoptotic events, thereby slowing the rate of age-related sarcopenia. This notion was supported by Radak and colleagues [10], who reported that levels of 8-oxoGua, following a short bout of exercise, quickly returned to pre-exercise levels in old trained but *not* sedentary individuals. Indeed, 8-oxoGua levels remained elevated in older sedentary subjects 24 hours after exercise. Radak [10] postulate that differential regulation of base excision repair (BER), could explain how trained old people are better able to withstand the genotoxic stress associated with ageing and exercise.

Poly(ADP-ribose) polymerase 1 (PARP-1) is a central mediator of the response to cellular stress caused by physiological stressors such as ROS, RNS and inflammation [19-21]. Indeed, PARP-1 is required for maintaining genome integrity and cellular homeostasis in response to oxidative stress [19,20]. It is therefore possible that poly(ADP-ribosyl)ation (PARylation) is differentially regulated in muscles from old active individuals compared with muscles from old, more sedentary people, although to date this hypothesis has not been tested. The present study, therefore, aimed to determine the influence of age and habitual activity level on (1) Protein levels of total PARP-1, cleaved PARP-1 (which is characteristic of apoptosis) and poly(ADP-ribose)glycohydrolase (PARG) (the protein responsible for degradation of PAR polymers) and (2) the effect of an acute exercise bout on these parameters. It was hypothesized that cleaved PARP-1 and PARG would be elevated at rest and following exercise in old persons with low physical activity levels but that life-long training would attenuate this phenomenon.

## Results

### Baseline data

#### Total PARP-1 protein content

**Figure 1:**
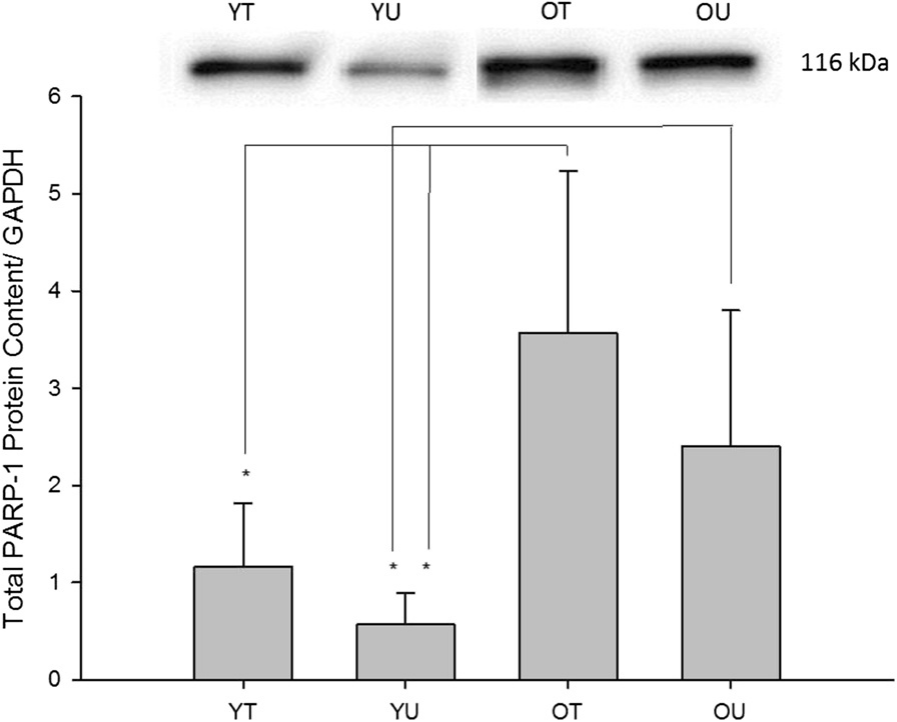
**Baseline total PARP-1 protein content measured in young trained (YT), young untrained (YU), old trained (OT) and old untrained (OU) participants.** Total PARP-1 protein content was significantly greater in OT compared with both young groups and was significantly greater in OU compared with YU. There was also a trend for OU being greater than YT (*P* = 0.086) * indicates significant difference (*P* < 0.05).

The effects of age and training status on baseline total PARP-1 can be seen in Figure 1. There was a significant difference in the baseline total PARP-1 between the four groups (*P* = 0.001). *Post-hoc* analysis confirmed that total PARP-1 was significantly increased in the old trained compared with both young trained (*P* = 0.001) and young untrained (*P* = 0.001) participants. Moreover, total PARP-1 was significantly increased in the old untrained compared with young untrained (*P* = 0.001) and there was a trend for an increase in the old untrained compared with young trained (*P* = 0.086). Taken together, these data suggest that regardless of training status, total PARP-1 protein content was greater in the old compared with the young participants.

#### Cleaved PARP-1 protein content

**Figure 2:**
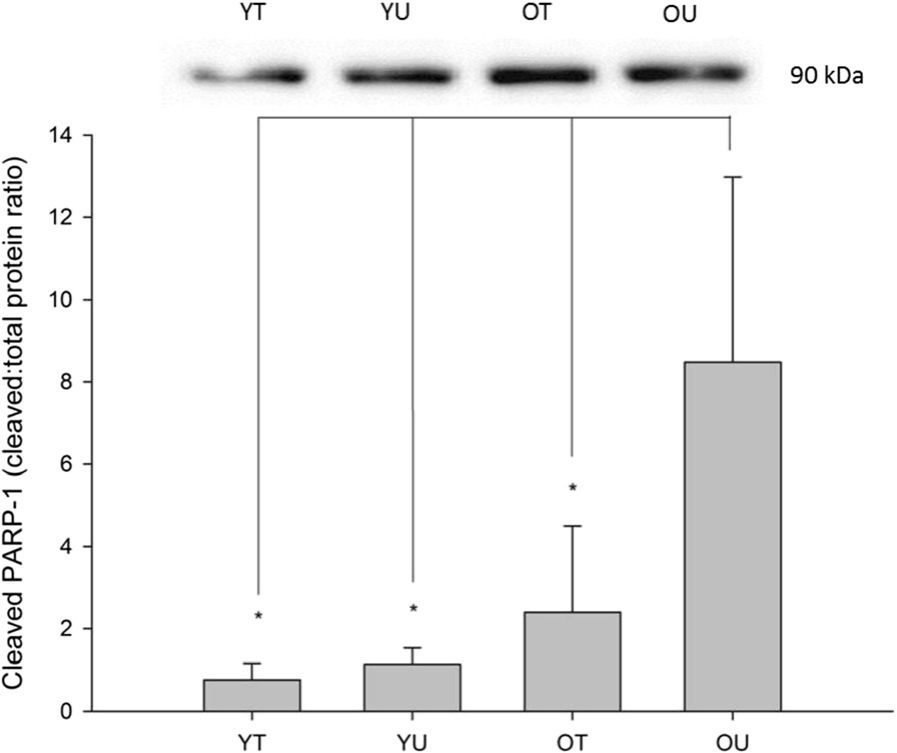
**Baseline cleaved PARP-1 protein content measured in young trained (YT), young untrained (YU), old trained (OT) and old untrained (OU) participants.** Cleaved PARP-1 protein content was significantly greater in OU compared with all other groups (*P* < 0.05). Interestingly, there was no significant difference between OT and either of the young groups. * indicates significant difference (*P* < 0.05).

The effects of age and training status on baseline cleaved PARP-1 can be seen in Figure 2. There was a significant difference in the baseline cleaved PARP-1 between the four groups (*P* = 0.046). *Post hoc* analysis confirmed that cleaved PARP-1 was significantly increased in old untrained subjects compared with old trained (*P* = 0.044), young trained (*P* = 0.013) and young untrained subjects (*P* = 0.018). Interestingly, there was no significant difference between old trained participants and either of the young groups (*P* > 0.05).

#### PARG protein content

**Figure 3:**
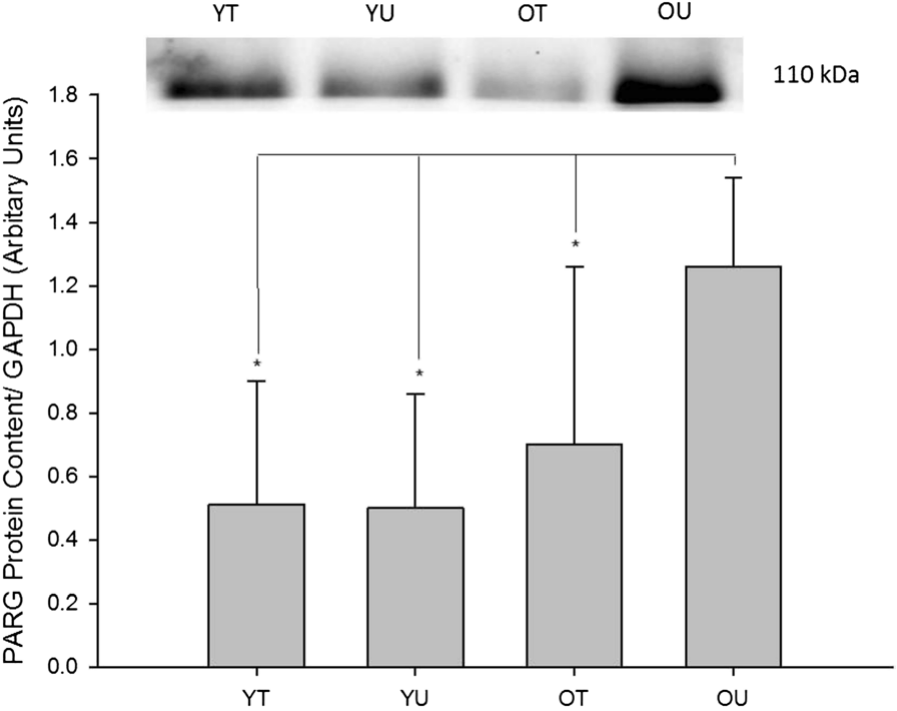
**Baseline PARG protein content measured in young trained (YT), young untrained (YU), old trained (OT) and old untrained (OU) participants.** PARG protein content was significantly greater in OU compared with all other groups (*P* < 0.05). Interestingly, there was no significant difference between OT and either of the young groups. * indicates significant difference (*P* < 0.05).

The effects of age and training status on baseline PARG, a negative regulator of PARP, can be seen in Figure 3. There was a significant difference in the baseline PARG protein content between the four groups (*P* = 0.016). *Post hoc* analysis confirmed that PARG was significantly increased in old untrained subjects compared with old trained (*P* = 0.031), young trained (*P* = 0.005) and young untrained subjects (*P* = 0.005). Interestingly, there was no significant difference between old trained participants and either of the young groups (*P* > 0.05).

### Exercise data

#### PARP-1 total protein content

**Figure 4:**
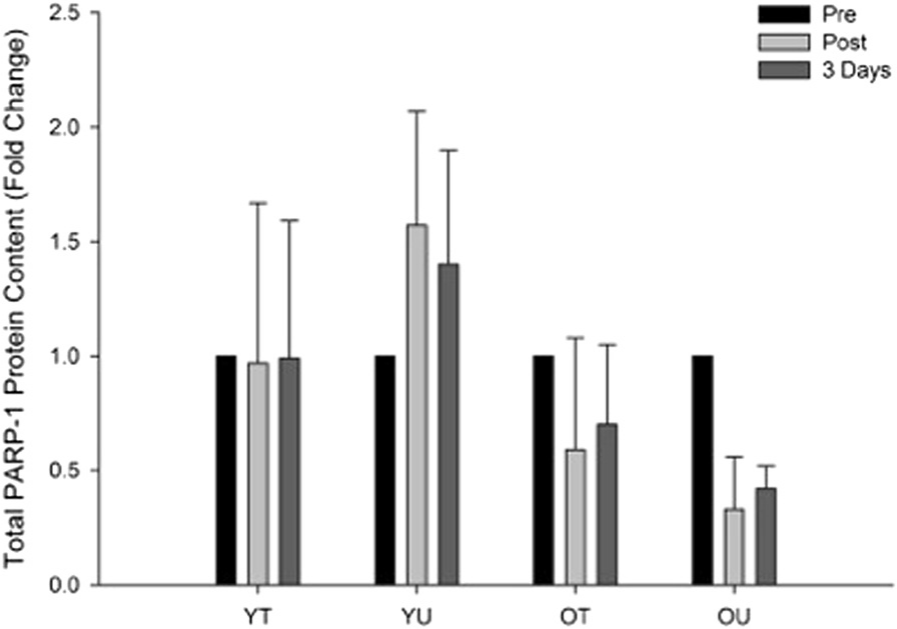
**The effects of HIT exercise on total PARP-1 protein content measured pre-exercise (Pre), immediately post-exercise (Post) and three days post-exercise in young trained (YT), young untrained (YU), old trained (OT) and old untrained (OU) participants.** There was a significant group by time interaction (*P* = 0.026) with total PARP-1 increasing following the exercise in the YU group whilst decreasing post-exercise in both OT and OU.

The effects of an acute bout of high-intensity interval (HIT) exercise on PARP-1 protein expression can be seen in Figure 4. There was a significant time x group interaction (*P* = 0.026) as well as main effects for group (*P* = 0.026) and time (*P* = 0.027). Exploration of the interaction suggested that there was no change in PARP-1 expression in the young trained group whilst there was an increase post and three days following exercise in the young untrained group. In contrast there was a decrease post and three days following exercise in both the old trained and untrained groups.

#### Cleaved PARP-1 protein content

**Figure 5:**
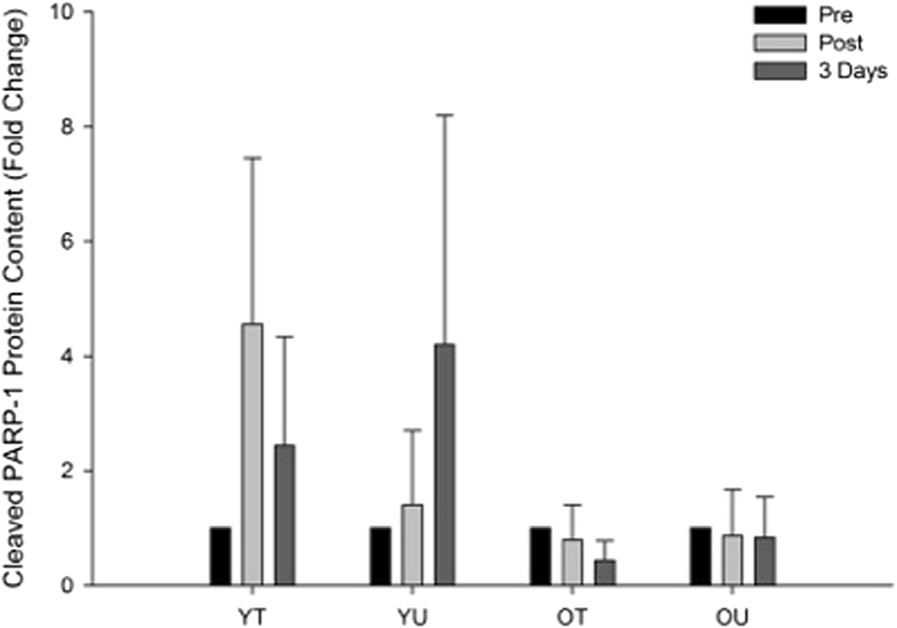
**The effects of HIT exercise on cleaved PARP-1 protein content measured pre-exercise (Pre), immediately post-exercise (Post) and three days post-exercise in young trained (YT), young untrained (YU), old trained (OT) and old untrained (OU) participants.** There was a trend for a group by time interaction (*P* = 0.072) with total PARP-1 increasing following the exercise in both young groups with no change in both old groups.

The effects of an acute bout of HIT exercise on cleaved PARP-1 protein expression can be seen in Figure 5. There was a trend towards a time x group interaction (*P* = 0.072) although there were no main effects for group (*P* = 0.134) or time (*P* = 0.505). This non-significant statistical trend appeared to suggest that cleaved PARP-1 protein expression increased immediately post and three days following exercise in younger but not older individuals, irrespective of training status.

#### PARG protein content

**Figure 6:**
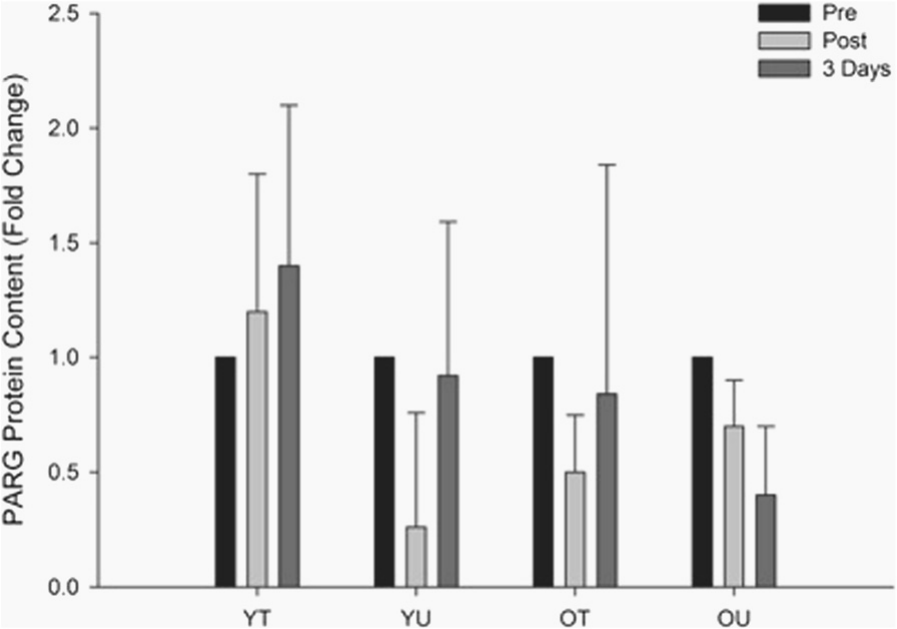
**The effects of HIT exercise PARG protein content measured pre-exercise (Pre), immediately post-exercise (Post) and three days post-exercise in young trained (YT), young untrained (YU), old trained (OT) and old untrained (OU) participants.** There was no group x time interaction (*P* = 0.139).

**Figure 7:**
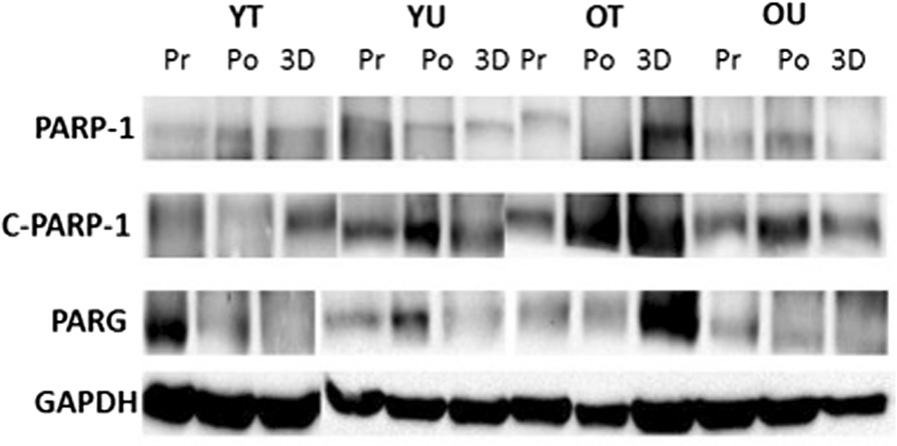
**Representative Western blot of PARP-1, cleaved PARP-1 (C-PARP-1) PARG and GAPDH (loading control) in young trained (YT), young untrained (YU), old trained (OT) and old untrained (OU) participants.** A representative blot is shown from pre-Exercise (Pr), post-exercise (Po) and three days post-exercise (3D).

The effects of an acute bout of HIT exercise on PARG protein expression can be seen in Figure 6. There was no time x group interaction (*P* = 0.139) or any main effects for group (*P* = 0.176) or time (*P* = 0.672). These data suggest that exercise did not affect PARG protein expression in any of the groups. Representative western blots for the exercise data can be seen in Figure 7.

## Discussion

The aim of the present study was to determine levels of PARP-1, cleaved PARP-1 and PARG at rest and following an acute exercise bout in order to investigate the effects of age and training status on indicators of genomic integrity and apoptotic pathways in human skeletal muscle. In this regard, we show for the first time that low physical activity levels in older people disrupts PARP-1 regulation in skeletal muscle leading to increased levels of PARP-1, a protein essential for recovery from DNA damage, and cleaved PARP-1, a characteristic of apoptosis. In support of our hypothesis, we report that lifelong training attenuates this deterioration in skeletal muscle. Additionally, we demonstrate that skeletal muscle ageing alters the response of total PARP-1 to an acute exercise bout. These findings provide novel insight into how habitual exercise training could protect the ageing genome, although further work is required to confirm this.

PARP-1 activity, predominantly by PARylation of both proteins and DNA, has key, direct and indirect, roles in the response to stress and repair of oxidative DNA damage [22,23]. PARP-1 PARylation is also implicated in signaling to the stress granules, the proteasome, in controlling the cellular localization of key transcription factors [19] and in cell senescence or programed cell death responses including apoptosis, necrosis and parthanatos [24]. Given the central role of PARP-1 in the repair of oxidative DNA damage and cell death decisions, it seemed possible that PARylation may be a key component of the protective effects of exercise. To examine this hypothesis, the levels of three proteins involved in the PARylation response to genotoxic stress, PARP-1, PARG and cleaved PARP-1 were measured. In young subjects at rest, irrespective of training, all of the three proteins were present at low levels. However, PARP-1 was significantly increased in all old subjects, irrespective of training status, presumably relating to the higher level of DNA damage in muscles from older participants [10]. Future studies should now investigate the exercise and age-related changes in post-translational modifications of PARP-1.

When apoptosis is initiated, PARP-1 is cleaved by apoptotic caspases, and its presence is considered a characteristic feature of apoptosis. However, PARP-1 can also be cleaved by other proteins, including apoptosis independent caspase 7 which cleaves PARP-1 located on the promoters of NFĸB target genes allowing expression of pro-inflammatory genes [25]. Although there has been some debate about the existence of caspase-dependent and independent apoptosis in skeletal muscle, it now seems likely that both pathways are indeed present [12,26,27]. Here, in young subjects at rest, the ratio of PARP-1:cleaved PARP-1 was low, perhaps indicating a low but not complete absence, of apoptotic and inflammatory related caspases. However, cleaved-PARP-1 was significantly elevated in the old untrained subjects, presumably indicating a highly stressed environment with myonuclear apoptosis and inflammation, compared with the much lower levels of cleaved PARP-1 observed in the trained old subjects. These data therefore suggest that exercise training slows, but does not abolish, the progress towards an age-related highly stressed state in skeletal muscle.

PARylation is NAD+ dependant, therefore regulation of PAR activity is vital to prevent depletion of NAD+ [28], which would affect other NAD+ dependant processes, such as the acetylation [29] and deacetylation (sirtuin based) pathways [30]. Consequently, a complex array of posttranslational and automodifications of PARP-1 [31] co-ordinate the multiple PARP-1 processes and are essential for maintaining NAD+ levels [32]. Depleted NAD+ levels could compromise metabolism and inhibit SIRT1, a key regulator of energy metabolism and mitochondrial biogenesis [33-35]. Inhibition of SIRT1 activity leads to mitochondrial dysfunction [30], mimicking the aberrant mitochondrial function and homeostasis seen in the muscles of sedentary aged humans [14,15,17]. PARG has a central role in the regulation of NAD+ levels, primarily via the NAD+ salvage pathway, but may also regulate the expression of PARP-1 [24]. In the present study, young participants had low levels of PARG irrespective of training status, although PARG levels were elevated in the old participants, especially the old untrained. The high PARP-1 levels seen in old untrained subjects may reflect high levels of DNA damage and will likely result in chronic PARylation causing depleted levels of NAD+. It is, therefore, proposed that the equally high PARG levels that were seen in these subjects represent an attempt to combat this PARylation-induced depletion in NAD+. The lower PARG levels in the old trained subjects may result from improved oxidative metabolism and mitochondrial biogenesis [36] counteracting the mitochondrial dysfunction and metabolic crisis associated with SIRT1 inhibition caused by NAD+ depletion [29].

We also investigated PARP-1, PARG and cleaved PARP-1 protein content immediately post and three days following an acute HIT exercise bout. A HIT model was employed due to this form of exercise being shown to be a tolerable, time-efficient, enjoyable and effective method of inducing metabolic adaptations in human skeletal muscle [36,37]. In addition, HIT activates both type I and type II muscle fibers [38,39], which is an important consideration when assaying a mixed fiber type muscle such as the vastus lateralis. Prior to the present study, PARP-1, PARG and cleaved PARP-1 had not been investigated in an exercise setting in humans, which consequently presented difficulties in selecting suitable biopsy time-points post-exercise. We elected to extract muscle samples at three days since our group has previously shown that stress-responsive proteins, such as heat shock proteins, are up-regulated at this time point [40,41]. We report that there were no significant changes in PARG levels immediately post and three days following exercise in any of the subject groups, suggesting that PARG is not responsive to an acute exercise bout. Interestingly PARP-1 levels showed no change in the young trained but increased in the untrained young. These data may suggest that in young subjects, habitual exercise provides protection against the potentially damaging, exercise-induced stress response [42]. However, in old subjects total PARP-1 expression decreased post-exercise, perhaps mediated by PARG repression of gene expression [24] and may be a further attempt to protect against NAD+ depletion. Gene expression and mRNA stability studies may provide further insight into this intriguing phenomenon. In young subjects, irrespective of training status, there was a trend towards an increase in cleaved PARP-1, which could indicate an increase in apoptosis following exercise but perhaps more likely reflects cleavage of PARP-1 at NFĸB target genes. In old subjects, there was a trend towards decreased cleaved PARP-1 expression following exercise which could account for the age-related attenuated induction of NFĸB stress response genes following muscle contraction [42]. Follow-up work should examine whether this trend reaches statistical significance in a larger subject cohort and aim to further investigate the underlying mechanisms.

## Conclusions

In conclusion, lifelong exercise training attenuates the increase in baseline cleaved PARP-1 and PARG expression that is observed in older individuals with low habitual physical activity levels. This novel finding suggests that life-long exercise training can slow down the progressive decline towards a chronically stressed environment in skeletal muscle, thus attenuating genomic instability. These data could have major implications for the prevention and treatment of sarcopenia and provide baseline data for future intervention studies.

## Methods

### Subjects

This study received institutional ethical approval from the Liverpool John Moores University ethical committee and adhered to the Declaration of Helsinki. After providing written informed consent, 24 male Caucasian subjects participated in this study. Subjects were allocated into four groups according to their age and training status, to yield four groups: young trained (*n* = 6); young untrained (*n* = 6), old trained (*n* = 6) and old untrained (*n* = 6). Baseline physical and physiological characteristics are shown in Table 1. Trained subjects were all competitive amateur cyclists that had habitually completed at least five endurance exercise sessions per week (all ≥ 45 minutes) as part of a systematic training regime. Old trained subjects had adopted such an exercise regime for a minimum of 10 years. Untrained subjects completed ≤ three non-endurance based exercise sessions per week. Verbal report and physiological assessment was utilized to verify the training history of our cohort.

**Table 1:** Mean (SD) characteristics of the four subject groups

	**Young trained**	**Young untrained**	**Old trained**	**Old untrained**	**Group *P *** **value**
Age (years)	24.2 (3.6)†	22.1 (3.4)†	64.5 (5.5)^a^	63.8 (3.0)^a^	0.001
Height (cm)	177 (8.6)	182 (6.5)	175 (6.8)	175 (9.3)	0.786
Mass (kg)	66.2 (10.0)	84.5 (10.8)	71.8 (7.8)	95.5 (24.8)^ab^	0.037
Lean mass and BMC (kg)	59.3 (6.3)	66.7 (8.9)	59.8 (7.4)	66.1 (6.8)	0.127
Fat mass (kg)	6.9 (1.5)	17.8 (2.1)^a^	12 (6.4)^a^	29.41 (12.3)^abc^	0.001
Body Fat (%)	10.5 (1.5)	21.1 (2.1)^a^	16.7 (1.8)^a^	30.8 (11.5)^abc^	0.001
VO_2max_ (ml.kg^-1^.min^-1^)	64 (7.0)	42 (4.1)^a^	47 (8.3)^a^	28 (9.8)^abc^	0.001

^a^denotes significant difference from young trained; ^b^denotes significant difference from young untrained; ^c^denotes a significant difference from old trained. BMC = bone mineral content.

### Physiological assessment

Maximal oxygen uptake (VO_2max_) and peak power output (PPO) were determined approximately one week prior to the main experimental trial, using an incremental exercise test performed until volitional exhaustion on a bicycle ergometer (Daum Electronic Ergo Bike, Daum, Germany). Oxygen uptake (Online Systems, Metamax Cortex,Germany) and heart rate (Polar S610i, Finland) were measured throughout the test. Following a five minute warm-up at 50 W, participants completed successive one minute exercise bouts with wattage being increased by 30 W every minute until volitional exhaustion. VO_2max_ was deemed to have been attained if the following criteria were met: 1) heart rate within 10 beats.min^-1^ of age-predicted maximum, 2) respiratory exchange ratio > 1.1, and 3) plateau of oxygen consumption despite increased workload. All subjects fulfilled these criteria.

### Intermittent exercise protocol

Subjects reported to the laboratory on the morning of the exercise trial after abstaining from exercise, alcohol and caffeine for 48 hours. Following a five minute warm-up at 50% PPO, a 20 minute HIT session was completed on a bicycle ergometer (Daum Electronic Ergo Bike, Daum, Germany). The HIT session consisted of a two minute bout at 40% PPO followed by a two minute bout at 80% PPO. This work-rest ratio was repeated five times. We chose to use the HIT model of cycling exercise since this form of exercise has been shown to be a tolerable, time efficient and effective method of inducting metabolic adaptations in skeletal muscle [36]. Oxygen uptake was recorded continuously using an online system (Metamax Cortex, Germany) whilst both heart rate (Polar S610i, Finland) and ratings of perceived exertion (RPE: Borg 6 to 20 scale) were recorded at two minute intervals.

### Muscle biopsies

Following the administration of a local anesthetic (0.5% marcaine), muscle biopsies were obtained from the vastus lateralis muscle using a Bard Monopty disposable biopsy instrument (12 cm x 10 cm gauge, Bard Monopty Systems, USA). Muscle biopsies were obtained at baseline, immediately post and three days following the exercise trial. The immediate recovery phase was assayed since we hypothesized that PARP-1 might be acutely up-regulated at this time-point owing to exercise-induced DNA damage. A late recovery biopsy time point was selected since our group had previously demonstrated that several stress-responsive proteins, namely heat shock proteins, are up-regulated three days following acute non-damaging exercise [40,41]. The same leg was utilized for all biopsies and biopsy sites were separated by at least three centimeters. Muscle samples (approximately 50 mg) were immediately snap frozen in liquid nitrogen and stored at −80°C for subsequent biochemical analysis.

### Western blotting

Approximately 20 to 30 mg of frozen muscle tissue was ground to powder and homogenized in 120 μl of ice cold lysis buffer that included phosphatase inhibitors (25 mM Tris/HCl (pH 7.4), 50 mM NaF, 100 mM NaCl, 5 mM EGTA, 1 mM EDTA, 10 mM sodium pyrophosphatase, 1 mM Na_3_VO_4_, 0.27 M sucrose, 1% Triton X-100, 0.1% 2-mercaptoethanol) and supplemented with a protease inhibitor tablet (Complete mini, Roche Applied Science, West Sussex, UK). Homogenates were centrifuged at 14,000 g for 10 minutes at 4°C before the protein content of the resultant supernatant was determined using a bicinchoninic acid assay (Sigma Aldrich, UK). Samples containing 50 μg protein were diluted with an equal volume of 2X Laemmli buffer (Geneflow Ltd, UK) and boiled for five minutes at 100°C. Samples were separated by molecular mass via SDS-PAGE using self-cast gels (Geneflow Ltd, UK) before being transferred semi-dry onto a nitrocellulose membrane (Geneflow Ltd, UK). For each blot, samples were run alongside a molecular weight marker (BioRad laboratories Ltd, UK) and negative control. Ponceau staining solution (Sigma Aldrich, UK) was used to verify successful gel to membrane protein transfer. Membranes were blocked for one hour at room temperature in Tris-buffered saline (TBST) with 5% non-fat dry milk (NFDM). Membranes were washed for three times five minutes in TBST before being incubated overnight at 4°C with antibodies for PARP-1 (Abcam, Cambridge, UK), cleaved PARP-1 (Abcam, UK) and PARG (Abcam, UK) all at concentrations of 1:1,000 in 1X TBST supplemented with NFDM. Membranes were probed for GAPDH (Cell Signalling, Cambridge, UK) to ensure equal protein loading between samples. Subsequently membranes were washed for three times five minutes in TBST before being incubated for 90 minutes with an appropriate secondary antibody. Following a further three times five minutes wash, membranes were exposed in a chemiluminescence liquid (SuperSignal, Thermo Fisher Scientific, Rockford, IL, USA) for two minutes and subsequently visualized using a Bio-Rad Chemi-doc system (BioRad laboratories Ltd, UK). Quantity One image-analysis (BioRad laboratories Ltd, UK) software was used to determine the intensities of protein bands.

### Statistical analysis

A one-way analysis of variance (ANOVA) was employed to analyze baseline differences between groups. If any significant F values were observed, least-significant difference (LSD) tests were performed to determine where any significant differences occurred. A two-way mixed design ANOVA was utilized to examine the interaction between group and exercise. An alpha value of *P* ≤ 0.05 was used for all tests and a statistical trend was defined as being less than twice the alpha value (that is, ≤ 0.1). All statistical analysis was performed with the statistical package for social sciences version 20.0 (SPSS, England). All data in text, tables and figures are presented as means (± SD).

## Abbreviations

PARP-1: Poly (ADP-ribose) polymerase-1; PARG: Poly(ADP-ribose) glycohydrolase; ROS: Reactive oxygen species; RNS: Reactive nitrogen species; BER: Base excision repair; HIT: High-intensity interval training; NAD+: Nicotinamide adenine dinucleotide; YT: Young trained; YU: Young untrained; OT: Old trained; OU: Old untrained.

## Competing interests

The authors declare that they have no competing interests.

## Authors’ contributions

JNC performed the majority of the laboratory work and drafted the manuscript. GS performed some of the Western blot analysis. SM, WG and SW performed the muscle biopsies and/or performed cardiac assessments on all the participants prior to allowing them to perform exercise trials. JGB and JPM participated in the design of the study and provided expert advice in the laboratory techniques. LAI and GLC conceived the study and edited the manuscript. All authors read and approved the final manuscript.
